# A universe of human gut-derived bacterial prophages: unveiling the hidden viral players in intestinal microecology

**DOI:** 10.1080/19490976.2024.2309684

**Published:** 2024-02-01

**Authors:** Zhangming Pei, Yufei Liu, Yutao Chen, Tong Pan, Xihao Sun, Hongchao Wang, R. Paul Ross, Wenwei Lu, Wei Chen

**Affiliations:** a State Key Laboratory of Food Science and Resources, Jiangnan University, Wuxi, P. R China; b School of Food Science and Technology, Jiangnan University, Wuxi, P. R China; c International Joint Research Center for Probiotics & Gut Health, Jiangnan University, Wuxi, Jiangsu, China; d APC Microbiome Ireland, University College Cork, Cork, Ireland; e National Engineering Research Center for Functional Food, Jiangnan University, Wuxi, P. R China

**Keywords:** Prophage, gut microbiome, host range, antibiotic resistome, virulence factor, gut phageome

## Abstract

Prophages, which are an existing form of temperate phages that integrate into host genomes, have been found extensively present in diverse bacterial species. The human gut microbiome, characterized by its complexity, dynamism, and interconnectivity among multiple species, remains inadequately understood in terms of the global landscape of bacterial prophages. Here, we analyzed 43,942 human gut-derived bacterial genomes (439 species of 12 phyla) and identified 105,613 prophage regions in ~ 92% of them. 16254 complete prophages were distributed in ~ 24% of bacteria, indicating an extremely uneven prophage distribution across various species within the human gut. Among all identified prophages, ~4% possessed cross-genera (2–20 genera) integration capacity, while ~ 17% displayed broad infection host ranges (targeting 2–35 genera). Functional gene annotation revealed that antibiotic-resistance genes and toxin-related genes were detected in ~ 2.5% and ~ 5.8% of all prophages, respectively. Furthermore, through sequence alignments between our obtained prophages and publicly available human gut phageome contigs, we have observed that ~ 72% of non-redundant prophages are previously unreported genomes, and they illuminate ~ 6.5–9.5% of the individual intestinal “viral dark matter”. Our study represents the first comprehensive depiction of human gut-derived prophages, provides a substantial collection of reference sequences for forthcoming human gut phageome-related investigations, and potentially enables better risk assessment of prophage dissemination.

## Introduction

Bacteriophages, the viruses that prey on bacteria, are prominent members of each microecosystem. Within the intricate tapestry of microbial life, a hidden world of genetic elements known as prophages is gradually being revealed. Prophages are not the conventional viral entities that propagate through lytic infection, but rather, they are essentially viral genomes that have integrated into the chromosomes of their bacterial hosts, adopting a clandestine existence. This integration has profound implications: protecting bacteria from superinfection by similar viruses,^1^ allowing the transfer of genetic material between bacteria,^2^ and providing selective advantages to the host.^3^ Moreover, prophages are pivotal players in the intricate microbial interaction network, impacting the composition and stability of microbial communities.^4^ Scanning the landscape of prophages is crucial for unraveling the complexities of microbial ecosystems and their impact on various fields, including medicine, biotechnology, and environmental science.

Improved bioinformatics tools and large-scale genome sequencing have greatly accelerated prophage mining. Researchers are engaged in the analysis of extensive bacterial genome datasets to effectively identify and categorize prophages across various species and environments.^5^ Certain pathogenic bacteria, such as *Helicobacter pylori*,^6^ *Clostridioides difficile*,^7^ *Salmonella enterica*,^8^ *Klebsiella pneumoniae*,^9^ *Streptococcus pneumoniae*,^10^ and *Mycobacterium*,^11^ have been demonstrated to harbor a wide array of prophages that exert an influence on host physiology, metabolism, and virulence. Nevertheless, the majority of existing prophage-related research has concentrated on a restricted range of bacterial species, resulting in a lack of comprehensive knowledge regarding the global diversity of prophages within the human gut.^12^ The human gut microbiome represents a complex, dynamic, and interconnected ecosystem comprised of multiple species, and a thorough exploration of bacterial prophages derived from the human gut will facilitate a deeper comprehension of the evolutionary dynamics of intestinal phages and shed light on bacteria-phage cross-kingdom interactions.

Aside from bacteria, the human gut also harbors a vast array of bacteriophages that exhibit specificity toward different bacterial hosts. This diversity contributes to the overall stability and resilience of the gut microbiome.^13^ Phages can influence the diversity of bacteria by selectively infecting certain species, which indirectly affects the composition of the entire microbial community.^14^ Virome or phageome analysis using a combined strategy of virus-like particle enrichment and metagenomic sequencing is becoming mainstream, which enables the detection of known viruses but also facilitates the study of previously unidentified viral entities without relying on culture.^15^ However, due to the lack of conserved genes representing genetic diversity similar to 16S in bacteria/archaea and 18S/ITS in fungi, the identification of the viral genome mainly relies on the alignments with the reference database.^16^ Multiple currently available human gut phageome/virome databases consist of assembled viral contigs obtained through next-generation metagenomics sequencing,^17–19^ and yield massive data from the perspective of independent viruses. Our objective is to collect concealed viral information from the perspective of bacterial isolates, which will instill greater confidence in uncovering the typically neglected “viral dark matter”.

**Figure 1.**
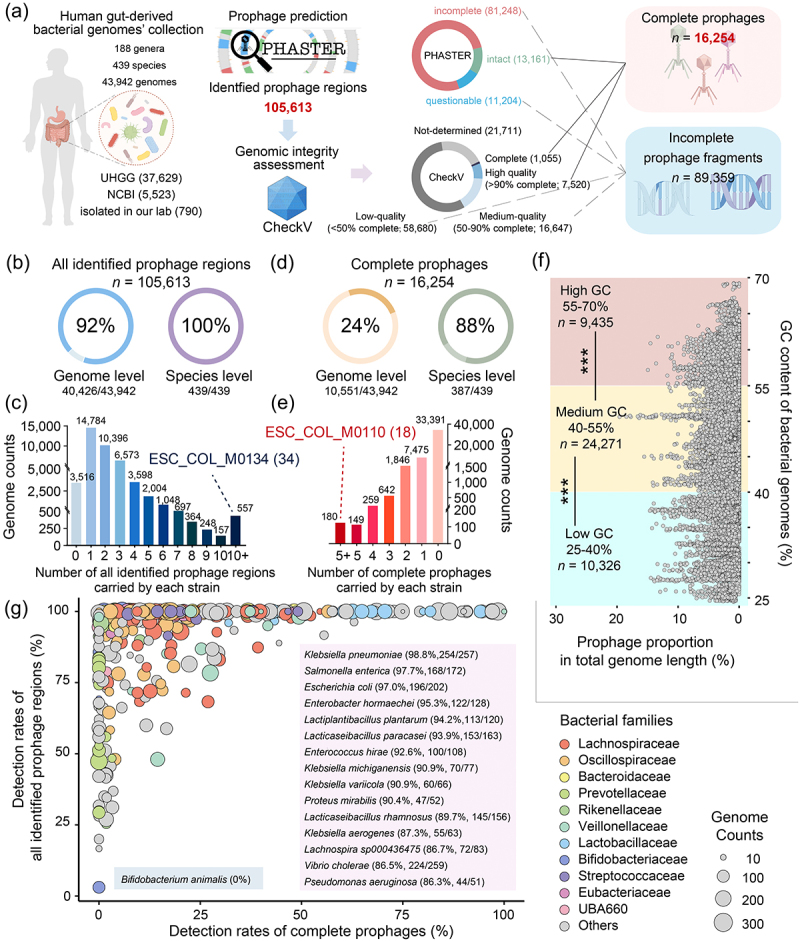
The overall landscape of human gut-derived bacterial prophages. (a) the brief pipeline of bioinformatics analysis in this study, including bacterial genomes’ collection, prophage prediction, genomic integrity assessment, and definition of related terms. (b) the detection rates of all identified prophage regions at the genome level and the species level. (c) the number of prophage regions carried by each strain. (d) the detection rates of complete prophages at the genome level and the species level. (e) the number of complete prophages carried by each strain. (f) the prophage gene contents of each strain. The bacterial genomes are divided into three groups based on their GC contents: the “low GC” group: bacterial genomes with GC content between 25–40%; the “Median GC” group: bacterial genomes with GC content between 40–55%; the “high GC” group: bacterial genomes with GC content between 55–70%. Significance tests were performed using the nonparametric Mann-Whitney U test, and the two-tailed *p* values were calculated. ***p < .001. (g) the detection rates of all prophage regions and complete prophages for each bacterial species. The size of the dot represents the number of genomes in the species and different colored dots represent different bacterial families. The specific data for each bacterial species is listed in Supplementary Table S4.

In this study, to deeply excavate the concealed viral components within the human gut and present a comprehensive prophage landscape, we conducted an extensive collection of 43,942 bacterial genomes derived from the human gut, representing 12 phyla, 20 classes, 34 orders, 65 families, 188 genera, and 439 species (Figure 1a and Supplementary Figure S1a). Subsequently, we ascertained the potential integration host range of all predicted prophages based on their sequence homology and determined their potential infection host ranges using the clustered regularly interspaced short palindromic repeats (CRISPR) system-spacer targeting approach.^20,21^ The complete presentation of the distribution of predicted antibiotic resistance genes (ARGs) and virulence factor-related genes (VFGs) located in bacterial prophage regions derived from the human gut was achieved through the annotation of all prophage genomes. Additionally, the alignments between our non-redundant prophages and the sequences of free phages within the human gut (human gut phageome data) allowed for the discovery of a large number of previously uncharacterized human gut-derived phages, as well as the facilitation of the identification of phage release by lysogenic bacteria within the intestinal environment. This study aims to shed light on the diversity, ecological, evolutionary, and functional significance of human gut-derived bacterial prophages, and inspire further exploration into this fascinating realm of microbial genomics.

## Results

### The overall landscape of prophage elements harbored in human gut-derived bacteria

Based on the prediction results of PHASTER (Figure 1a), our analysis illustrated the presence of 105,613 prophage regions (median length: 15.2 Kb, interquartile range [IQR]: 9.2–25.4 Kb), which were identified in ~ 92% of the genomes and completely covered the entirety of the 439 bacterial species included in our database (Figure 1b). The number of identified prophage regions varied greatly among the strains (Figure 1c): approximately one-third of strains harbored only one prophage, while 557 strains carried more than 10 prophages. It is a wonder that 34 prophages were detected in the genome of *Escherichia coli* strain ESC_COL_M0134. Given that a considerable portion of the predicted prophage regions were probably incomplete prophage fragments, we attempted to filter them out and set up a subset comprising 16,254 complete prophages (evaluated as “intact” by PHASTER or as “complete/high quality” by CheckV). It can be observed that complete prophages were distributed in ~ 24% (*n* = 10,551) of all bacterial genomes and ~ 88% of the bacterial species (Figure 1d). Out of these 10,551 bacteria that detected complete prophages, the vast majority of them carried a maximum of two complete prophages (Figure 1e). However, there are several bacterial hosts parasitized by multiple complete phages that cannot be ignored; especially in the genome of another *E. coli* strain, ESC_COL_M0110, as many as 18 complete prophages were identified. The huge variation in the number of prophages underscores the highly uneven prophage distribution among human gut-derived bacteria. Moreover, we also estimated the prophage gene content within each genome, but in this regard, there is little difference between various bacterial strains (median: 1.1%, IQR: 0.5–2.1%). Nevertheless, the prophage contents in the genomes of a handful of strains can reach up to 20% (Figure 1f; Supplementary Table S2).

The extent of variations in Guanine and Cytosine (GC) content among prokaryotes is considerable, exerting influences on species ecology, distribution, environmental adaptation, and lifestyle.^22^ Out of an interest in whether there is a possible linkage between the GC content of bacterial genomes and the integration of prophages 43,942 human gut-derived bacteria were divided into the “Low GC” group (25%–40%; *n* = 10,326), the “Medium GC” group (40%–55%; *n* = 24,271), and the “High GC” group (55%–70%; *n* = 9,435); subsequently, a comparison was made regarding the differences in prophage gene content and the number of prophages among these groups. It is indicated that there is a notable tendency for bacteria with high GC content to possess a significantly lower prophage gene content (*p* < .001; Figure 1f) and a smaller number of prophages within their genomes (*p* < 0.001; Supplementary Figure S1b,c), whereas bacteria with low GC content exhibit the opposite trend.

In addition, we specifically focused on the prophage incidence in distinct bacterial species. As shown in Figure 1g, prophage regions were detected in all strains of 207 bacterial species. The occurrences of complete prophages in 15 species are greater than 85%; of them, three (conditioned) pathogenic bacterial species, *K. pneumoniae* (98.8%), *S. enterica* (97.7%), and *E. coli* (97.0%) lead all other species. It is worth mentioning that the probabilities of certain commonly employed probiotic species harboring complete prophages are extremely high: *Lactiplantibacillus plantarum*–94.2%, *Lacticaseibacillus paracasei*–93.9%, and *Lacticaseibacillus rhamnosus*–89.7%. Not only that, *Lactococcus lactis*, *Limosilactobacillus reuteri*, *Ligilactobacillus salivarius*, and *Lactobacillus gasseri* also manifest a higher prophage content (Supplementary Fig. S1D). In contrast, prophage integration is extremely rare in another widely used probiotic species, *Bifidobacterium animalis*, whether complete prophages or prophage regions (Figure 1g). For some species in the genera *Bacteroides*, *Phocaeicola*, *Bifidobacterium*, *Alistipes*, and *Akkermansia*, while prophages are present in nearly all strains, complete prophages are scarce, with almost all of them being incomplete prophage fragments (Supplementary Figure S1E). That is, some symbiotic bacterial species that are generally advantageous in the healthy human gut appear capable of domesticating bacteriophages into cryptic prophages that cannot be released through lysogeny.^23^

### Approximately 4% of prophages possess the capacity to integrate across distinct bacterial genera in the human gut

**Figure 2.**
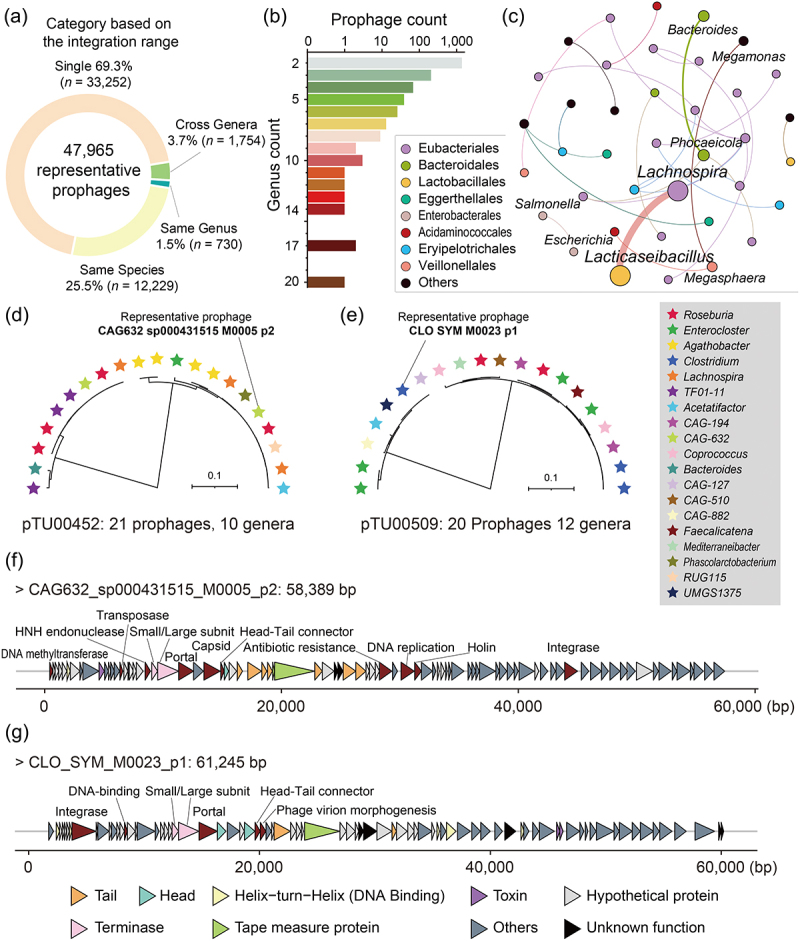
The integration host range of human gut-derived bacterial prophages. (a) 47965 representative prophages were classified into four categories based on their integration host range. Single: this prophage is only integrated into a single bacterial genome; same Species: this prophage can be integrated into different genomes of the same bacterial species; same Genus: this prophage can be integrated into genomes of different bacterial species within the same bacterial genus; Cross Genera: this prophage can be integrated into genomes of different bacterial bacterial genera. (b) the number of mapped bacterial genera for each cross-genera integrated prophages. (c) integrating associations between host genera for each cross-genera integrated prophages. The size of dots and the thickness of lines represent the strength of interactions. Different colored dots represent different bacterial families. The clustering details of all prophages are listed in supplementary table S5. (d,e) clustering and phylogenetic trees of pTU00452 and pTU00509. Different colored stars represent different host bacterial genera. (f,g) genomic organizations of the representative phages CAG632_sp000431515_M0005_p2 (pTU00452) and CLO_SYM_M0023_p1 (pTU00509). Each gene is colored based on its known or putative function.

With a strong focus on the ecological landscape of prophage integration, understanding whether they spread between different bacterial hosts will be essential for revealing the critical phage-bacteria-human host tripartite interactions. Clustering all 105,613 identified prophages based on their pairwise genomic similarities (>90% identity and > 90% query coverage) into prophage operational taxonomic units (pTU), we retained a total of 47,965 representative prophages (Supplementary Table S5). Of them, ~70% were observed as singletons, exclusively integrated within unique strains; ~25% were detected in the genomes of diverse bacterial strains belonging to the same bacterial species; whereas ~ 4% (*n* = 1,754) exhibited the capacity to integrate across various bacterial genera (Figure 2a). The majority of instances involving prophages capable of integrating across genera consist of only two distinct genera (Figure 2b), and the most prevalent cross-genera prophage integration is observed between the genera *Lacticaseibacillus* and *Lachnospira*, with 406 pairs involved (Figure 2c). This finding suggests that commonly used lactobacilli may pose a remarkably high risk of prophage transmission in the human gut. Furthermore, the occurrence of prophage transmission between *Escherichia* and *Salmonella* (6 pairs) raises concerns about gene exchange between pathogens of different genera facilitated by prophages.

Although cross-genera integration is not a common phenomenon in massive prophages, the presence of ultra-broad-spectrum prophages cannot be neglected. In our dataset, we have identified a total of 10 prophages capable of integrating into the genomes of 10 or more distinct bacterial genera (Figure 2d,e, Supplementary Figure S2a–h). Among them, the representative prophages of pTU00452 (CAG632_sp000431515_M0005_p2; Figure 2f) and pTU00509 (CLO_SYM_M0023_p1; Figure 2g) exhibit the most comprehensive structure, encompassing all genes associated with the core functional modules of bacteriophages: tail protein, head fiber, portal protein, major capsid protein, tape measure protein, terminase small and large subunits, integrase, and holin. Notably, the occurrence of the helix-turn-helix (HTH) protein in these ultra-broad-spectrum prophage genomes is much higher; specifically, CLO_SYM_M0023_p1 harbors 10 genes encoding HTH protein in its genome, surpassing other prophages in this regard (Figure 2g and Supplementary Figure S3a). Following phage adsorption and subsequent injection of their DNA, HTH proteins primarily serve to bind bacterial chromosomes, which might be a contributing factor in helping these phages integrate themselves into the genomes of numerous bacterial genera. Furthermore, our findings indicate that 13 prophages are highly prevalent (>75% of strains) within the same bacterial species (Supplementary Figure S3b); pTU00031 (PAPRE_XYL_M0027_p1) stands out as it is not only universally distributed among all *Paraprevotella xylaniphila* strains but also capable of detecting multiple copies within a single genome.

### Phage-bacteria linkage reveals the potential infection range of human gut-derived bacterial prophages

**Figure 3.**
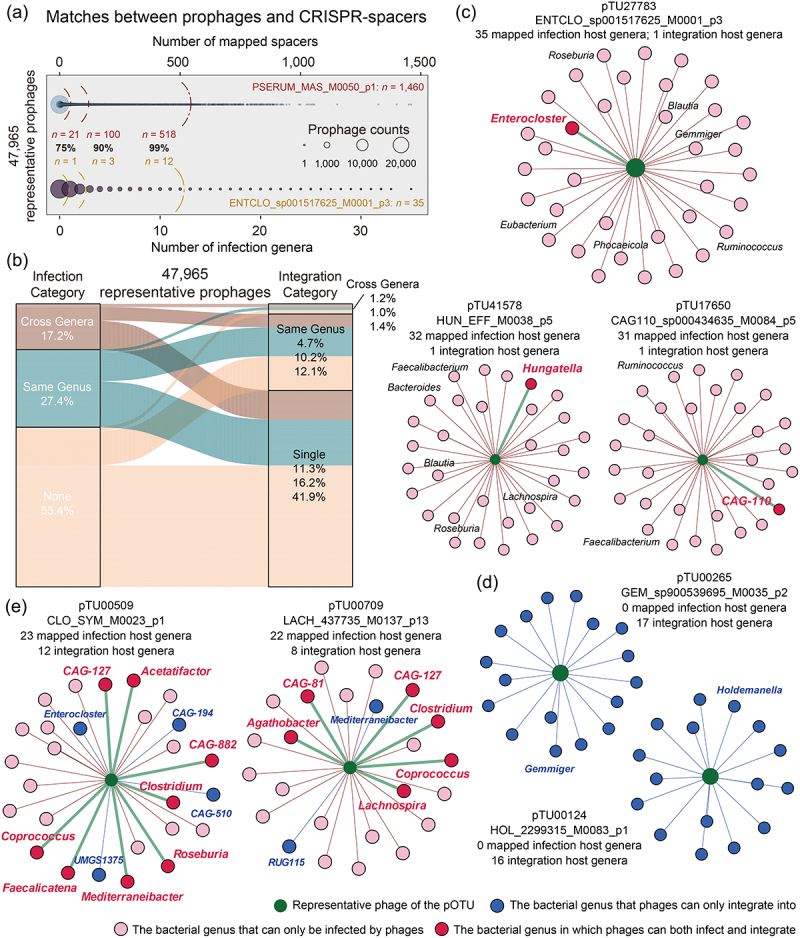
The infection host range of human gut-derived bacterial prophages. (a) the number of mapped CRISPR-spacers and the number of mapped infection host genera for each prophage. The three circular arcs from left to right represent the number of spacers and host genera matched with the top 25%, 10%, and 1% of the phages. (b) the Sankey diagram shows the classification of all 47,965 prophages by infection range and integration range. (c) the infection host range and integration host range of the “super killers”: pTU27783, pTU41578, and pTU17650. (d) the infection host range and integration host range of the “hitchhikers”: pTU00265 and pTU00124. (e) the infection host range and integration host range of “the mighty one”: pTU00509 and pTU00709. The green dots represent phages; the pink dots represent the bacterial genera that can only be infected by phages; the blue dots represent the bacterial genera that phages can only integrate into; and the red dots represent the bacterial genera in which phages can both infect and integrate into.

The infection pattern of bacteriophages typically exhibits a dynamic conversion between lysogenic and lytic cycles. Prophages could be induced and assembled into fully phage particles under certain pressure conditions, which can potentially disrupt the homeostasis of the gut microbiome. Consequently, in addition to emphasizing horizontal gene transfer facilitated by prophages, it is imperative to pay particular attention to their infection host ranges. The presence of spacers in CRISPR systems allows us to trace the infecting history of phages, and we successfully identified the putative infection bacterial hosts on the genus level for ~ 45% of prophages using the CRISPR-spacer targeting approach (Supplementary Table S6). It is illustrated that ~ 20% of prophages targeted 1–20 spacers, ~14% were linked to 21–100 spacers, and ~ 1% were paired with > 100 spacers (Figure 3a). Notably, the phage pTU18516 (PSERUM_MAS_M0050_p1) exhibited the highest number of recorded infections (*n* = 1,460). Among all spacer-targeted prophages, 27.4% were found to be host-restricted (targeting a single bacterial genus), while the remaining 17.2% displayed a broad host range (targeting 2–35 distinct bacterial genera). Compared to relatively rare cross-genera integration (~4%), the proportion of phage cross-genera infection is much higher.

The linkages between the infection host range and integration host range for all prophages revealed an impressive bacteria-phage conflict in the human gut. As shown in Figure 3b, ~41.9% of prophages are “Silencers”, exclusively integrated into the genome of a single human intestinal bacteria, and have not left immune records in the defense system of any strain. ~11.3% are “Killers” – they possess the capacity to infect a wide range of bacterial hosts across different genera. In particular, phages pTU27783 (ENTCLO_sp001517625_M0001_p3), pTU41578 (HUN_EFF_M0038_p5), and pTU17650 (CAG110_sp000434635_M0084_p5) are “Super Killers” that have previously infected 35, 32, and 31 different bacterial genera, respectively (Figure 3c), and it is noteworthy that the accidental integration of these phages was observed solely in a singular bacterial strain. It is implied that these phages prefer to battle rather than compromise with bacteria. ~1.4% of the prophages are “Hitchhikers” – they could move between the genomes of multiple bacteria; however, it is evident that these phages exclusively engage in the lysogenic cycle and hardly undergo the lytic cycle (Figure 3d). An additional 1.2% are “the Mighty Ones”, such as the phages pTU00509 (CLO_SYM_M0023_p1) and pTU00709 (LACH_437735_M0137_p13), which both exhibited a wide range of infection capabilities and integration potential (Figure 3e). It appears that these phages could freely switch infection modes depending on the bacterial hosts and the prevailing environmental pressures. Overall, the comprehensive scanning of the infection and integration host ranges of prophages outlines a global pattern of phage-bacteria interaction in the human gut environment.

### Mobile antibiotic resistome of human gut mediated by prophages

**Figure 4.**
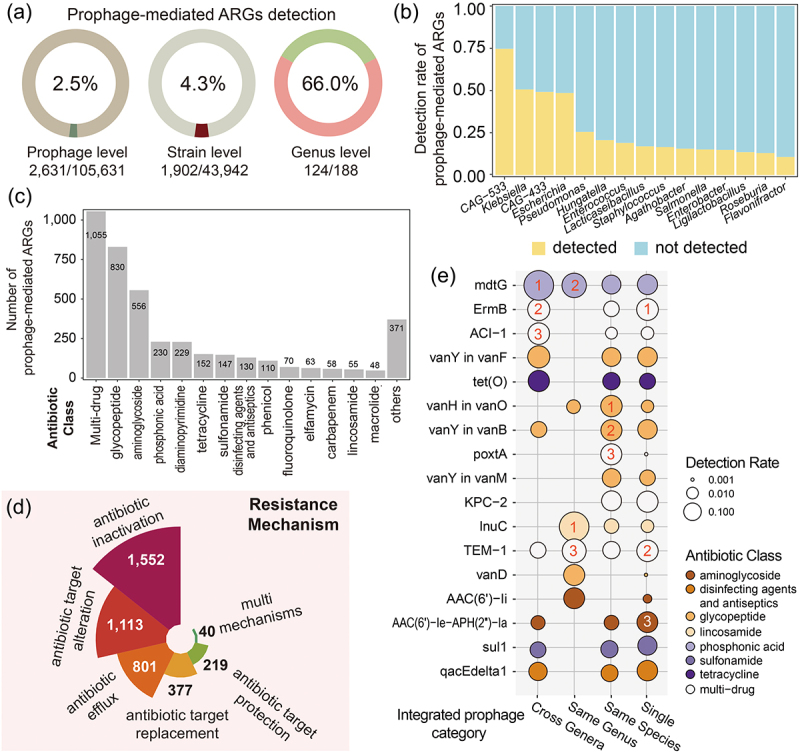
The antibiotic resistome of human gut-derived bacterial prophages. (a) the detection rates of prophage-mediated ARGs at the prophage level, the bacterial strain level, and the host genus level. (b) the top 10 bacterial host genera with the highest detection rate of prophage-mediated ARGs. (c) the antibiotic classes of prophage-mediated ARGs. (d) the resistance mechanisms of prophage-mediated ARGs. (e) the detection rates of various ARGs in prophages with different integration host ranges. The numbers on the circle represent the ranking of ARG detection rates in each group.

As one of the important carriers of horizontal gene transfer, prophages, through integration, can disseminate resistance genes within bacteria, facilitating the rapid acquisition of new resistance by the host when confronted with antibiotic pressure or other adverse conditions. Therefore, a comprehensive understanding of the distribution of resistance genes in prophage genomes contributes to the comprehension, surveillance, and effective control of the dissemination of antibiotic resistance in intestinal bacteria. We observed 4,104 potential ARGs in 2,631 (~2.5%) prophages involving 1,902 (~4.3%) bacterial genomes and ~ 66.0% genera in the catalog (Figure 4a and Supplementary Table S7). *CAG-533* was the most frequently detected genus, with ~ 74.7% (56/75) of bacteria found to harbor prophages carrying ARGs (Figure 4b), followed by *Klebsiella* (50.6%, 274/541), *CAG-433* (49.1%, 28/57), and *Escherichia* (48.5%, 98/202). It is worth noting that the genera *Lacticaseibacillus* (17.0%, 54/319) and *Ligilactobacillus* (13.5%, 46/341) also exhibited significant involvement in the detection of prophage-mediated ARGs, suggesting that prophages may serve as important vectors for ARGs in lactobacilli.

We identified a total of 301 prophage-mediated ARGs (Supplementary Figure S4A), encompassing a wide range of major antibiotic classes commonly employed in healthcare centers and the animal industry. Among these ARGs, those conferring resistance to multi-drug (*n* = 1,055), glycopeptide (*n* = 830), and aminoglycoside (*n* = 556) were found to be the most prevalent, followed by genes conferring resistance to phosphonic acid (*n* = 230), diaminopyrimidine (*n* = 229), tetracycline (*n* = 152), and sulfonamide (*n* = 147) (Figure 4c). The predominant resistance mechanisms observed in human gut-derived bacterial prophages included antibiotic inactivation, alteration of antibiotic targets, efflux pump activity, target replacement, cellular protection, and other multifaceted mechanisms (Figure 4d). While there were no significant variations observed in the quantity and occurrence rate of ARGs among prophages with different integration host ranges (Supplementary Figure S4b), notable distinctions were found in the specific types of resistance genes they harbored (Figure 4e). The integrated prophages spanning multiple genera primarily contained the core ARGs *mdtG* (53%; fosfomycin), *ermB* (9%; erythromycin), and *ACI-1* (7%; cephalosporin, penam, and penem), whereas the prophages restricted to a single host genus predominantly carried the core ARGs *lnuC* (51%; lincosamide), *mdtG* (15%), and *TEM-1* (13%; monobactam, cephalosporin, penam, and penem). It is also found that the presence of ARGs in single species-restricted prophages and prophages unique to individual strains exhibits greater diversity compared to the detection of specific ARGs in broad integration range prophages, with each gene being detected at a frequency of less than 10%. In conclusion, our analysis of the antibiotic resistome reaffirms that it is imperative to pay attention to ARGs carried by cross-genera integrated prophages or lactobacilli prophages, thereby thoroughly assessing their potential transfer risks in the human gut.

### Over half of the prophages encode various potential virulence factor genes

**Figure 5.**
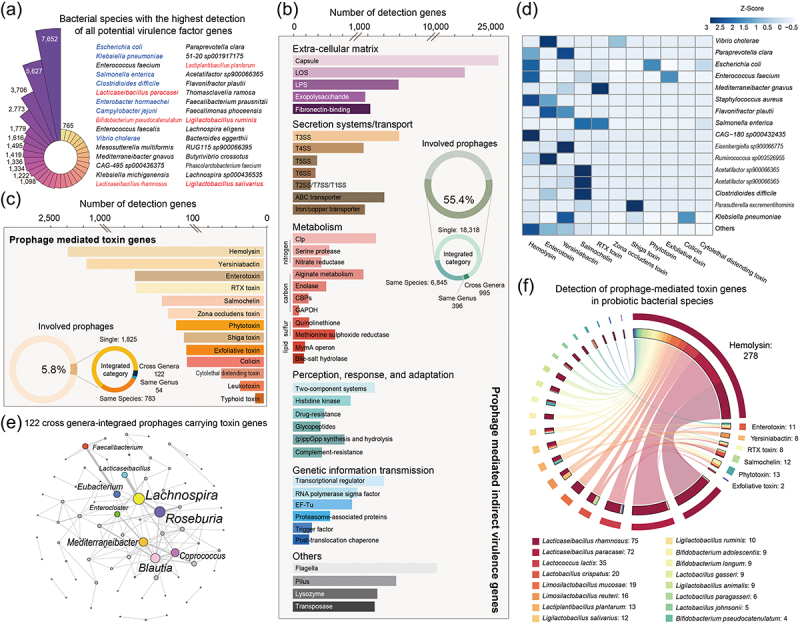
The prediction of virulence factor genes among human gut-derived bacterial prophages. (a) bacterial species with the highest detection of all potential VFGs. The captions are arranged in order of detection frequency from top to bottom and from left to right. The bacterial species in blue font are (conditional) pathogenic bacteria and the bacterial species in red font are probiotics. (b) the number of detected prophage-mediated indirect VFGs. (c) number of detected prophage-mediated toxin VFGs. The circles represent the VFGs detection frequencies of prophages with different integration host ranges. (d) the heatmap illustrates the bacterial species with the highest detection of various prophage-mediated toxin VFGs. (e) the network displays the host interactions of 122 cross-genera integrated prophages with detected toxin VFGs. (f) detection of prophage-mediated toxin genes in probiotic bacterial species.

Prophages, being a crucial vector of horizontal gene transfer, are not only involved in the dissemination of ARGs but also in the advancement of host pathogenicity by imparting VFGs to bacteria. To gain a comprehensive comprehension and ensure efficient surveillance of the transmission risk of VFGs in human gut-derived bacteria via prophages, we conducted a thorough examination of all 47,965 prophage genomes in our dataset, resulting in the identification of 138,176 potential VFGs (Supplementary Table S8). It is not surprising that pathogenic or conditionally pathogenic bacterial species with higher frequencies of prophages, namely *E. coli* (*n* = 7,562), *K. pneumoniae* (*n* = 5,627), *S. enterica* (*n* = 2,773), and *C. difficile* (*n* = 1,779), exhibited a greater frequency of detecting prophage-mediated VFGs compared to other species within the intestinal commensal microbiota (Figure 5a). Furthermore, certain species of lactobacilli and bifidobacteria also demonstrated a higher presence of prophage-mediated VFGs. However, our findings indicated that the majority of the identified potential VFGs acted in a manner that suited the host’s capacity for colonization, reproduction, and dissemination rather than exerting a direct impact on the human body. These virulence traits could be categorized into the terms cell-extracellular matrix, secretion machinery, substance metabolism, regulators, genetic information transmission, etc. (Figure 5b). These genes were observed to be distributed within 55% of prophages, implying that the integration of prophages to confer a competitive advantage to the host is relatively widespread in the human gut. In addition, there was a minimal disparity in the occurrence of indirect VFGs among prophages with varying integration host ranges (strain-specific: 55.1%; species-specific: 56.0%; genus-specific: 54.2%; cross-genera: 56.7%).

Given the ability of exotoxins to elicit various host responses through direct interaction with cell receptors or enzymatic modulation, our subsequent focus will be on VFGs encoding toxins. A total of 6139 toxin-associated VFGs, which belonged to 14 major classes, were identified in 5.8% of prophages (Figure 5c). Among these, hemolysins-related VFGs were found to have the highest occurrence, primarily in prophages of species *CAG-180 sp000432435*, *Staphylococcus aureus*, and *Enterococcus faecium*; followed by yersiniabactin, enterotoxin, RTX toxin, and salmochelin, which exhibited the highest occurrence in prophages of species *Eisenbergiella sp900066775*, *Ruminococcus sp003526955*, *Acetatifactor sp900066365*, and *Mediterraneibacter gnavus*, respectively (Figure 5d). Within all the prophages that detected toxin VFGs, the detection frequency of cross-genera integrated prophages (7%) was found to be higher than the average, and more importantly, this phenomenon involved several cornerstone genera of the human gut microbiota, including *Lachnospira*, *Roseburia*, *Blautia*, *Eubacterium*, and *Faecalibacterium* (Figure 5e). Not only that, our analysis revealed a concerning total of 332 exotoxin-associated VFGs mediated by prophages were identified within the genomes of 323 bacterial strains belonging to 16 commonly utilized probiotic species (Figure 5f), of which the species *L. rhamnosus* and *L. paracasei* played vital roles in toxin gene spreading through cross-genera prophage integration, further emphasizing their risks in this context.

### Excavating prophages illuminates a portion of “viral dark matter” in the human gut

**Figure 6.**
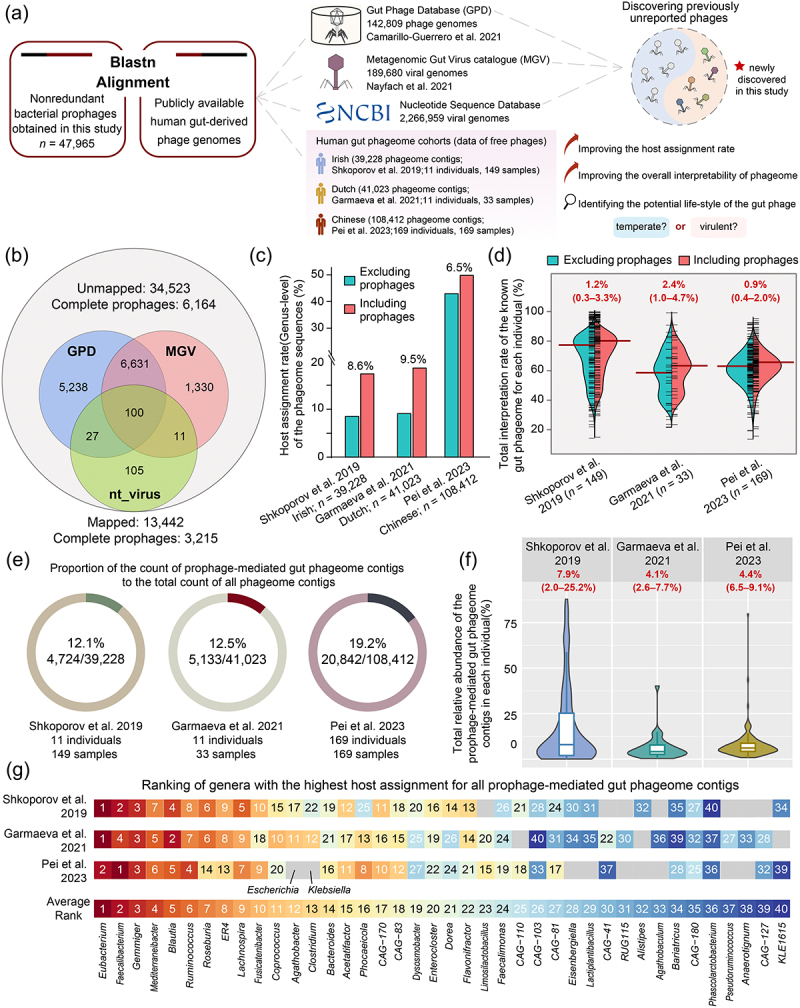
The linkages between human gut-derived bacterial prophages and human gut phageome contigs. (a) the brief analysis pipeline of sequence alignments between human gut-derived bacterial prophages and publicly available human gut phages. (b) the Venn diagram shows that out of the 47,965 prophages genomes in our dataset 13,442 generated a sufficient alignment with known genomes from GPD, MGV, or NCBI virus databases, and an additional 34,523 were newly discovered. The specific results are listed in supplementary Table S9. (c) the host assignment rates at the bacterial genera level of the human gut phageomes in three studies. (d) the total interpretation rates of the known gut phageomes for each individual in three studies. The green section represents the numerical values before incorporating our prophages, while the red section represents the numerical values after incorporating our prophages. (e) the circle charts illustrate the proportions of the count of prophage-mediated gut phageome contigs to the total count of all phageome contigs in three studies. (f) the total relative abundances of the prophage-mediated gut phageome contigs for each individual in three studies. (g) rankings of bacterial genera with the highest host assignment for all prophage-mediated gut phageome contigs in three studies. The overall ranking is based on the average ranking of three studies.

Human gut virome or phageome analyses, where viruses or phages are detected and identified by metagenomic sequencing, are becoming mainstream but constrained by the lack of reference sequences. Two recently released large-scale human gut phageome/virome databases, the Gut Phage Database (GPD) and the Metagenomic Gut Virus Catalog (MGV), have boosted research, increasing confidence in uncovering “viral dark matter”. To determine the contribution of our dataset to the human gut bacteriophage database, all 47,965 non-redundant prophage genomes were compared against the sequences in the GPD, the MGV, and the National Center for Biotechnology Information (NCBI) GenBank virus database (Figure 6a). In total, only ~ 28% (*n* = 13,442) of prophages in our dataset generated sufficient alignments with known phages, whereas the remaining ~ 72% are the previously unreported phage genomes, of which 6,164 are complete prophages (Figure 6b). These newly discovered high-quality gut-derived bacterial prophage genomes greatly expand the reference genome database of human gut phageomes.

Subsequently, to test whether the human gut-derived bacterial prophages obtained from the present study could enhance the recognition of human gut phageomes, we retrieved the representative human gut phageome contigs from the longitudinal Irish study (*n* = 39,228; 149 samples from 11 individuals), the longitudinal Dutch study (*n* = 41,023; 33 samples from 11 individuals), and the cross-sectional Chinese study (*n* = 108,412; 169 samples from 169 individuals) (Figure 6a). A unified approach (see Methods) was applied to assign bacterial hosts for phageome contigs in three studies. The original host assignment rates at the bacterial genus level of the phageome contigs in Irish, Dutch, and Chinese studies were 8.9%, 9.4%, and 42.4%, respectively, and after pooling our prophage sequences into the reference database, the rates increased by 8.6%, 9.5%, and 6.5%, respectively (Figure 6c). However, these newly annotated phageome contigs did not mark a prominent advance in the total interpretation rate of the known gut phageome for each individual; the total number of reads mapped from viral metagenomics of each sample in the Irish, Dutch, and Chinese studies only increased by a median of 1.2% (0.3–3.3%), 2.4% (1.0–4.7%), and 0.9% (0.4–2.0%), respectively (Figure 6d). Out of interest in the proportion of phages released from prophages in human gut phageomes, we performed pairwise genome alignments between all phageome contigs of the three studies and all prophages to assume the possible lifestyle of each phage in the human gut. The results showed that there were at least 12.1%, 12.5%, and 19.2% of prophage-mediated phages in human gut phageomes in Irish, Dutch, and Chinese studies, respectively (Figure 6e). Nevertheless, the total relative abundances of the prophage-mediated gut phageome contigs in each individual of the three studies were 7.9% (2.0–25.2%; Irish), 4.1% (2.6–7.7%; Dutch), and 4.4% (6.5–9.1%; Chinese) (Figure 6f). Our observations suggest that the phages released by lysogenic bacteria might only account for a small portion of the entire human gut phageome. The main contribution of our newly discovered human gut bacterial prophages was that they boosted the mining of non-predominant phages in the human gut.

In addition, we have counted the hosts of human gut phageome contigs that match the prophages, aiming to identify the lysogenic bacterial genera that are more prone to prophage release in the human gut. The combined findings from three independent studies revealed that the most frequently detected bacterial genera were *Eubacterium*, *Faecalibacterium*, *Gemmiger*, *Mediterraneibacter*, *Blautia*, *Ruminococcus*, and *Roseburia* (Figure 6g). Meanwhile, other human intestine cornerstone bacterial genera, such as *Clostridium*, *Bacteroides*, and *Phocaeicola*, were also identified as important “prophage releasers” (13^th^, 14^th^, and 16^th^ rank, respectively). However, upon closer examination of each study, it was observed that the two European studies exhibited greater similarity in the detection patterns of prophage-mediated phages, whereas the Chinese study displayed some minor deviations from them (*Faecalibacterium* ranked first; *Escherichia* and *Klebsiella* ranked in the top 15). Given the substantial influence of geographical factors on the human gut bacteriome, it is plausible that the observed deviation may be attributed to variations in the bacteriome composition among the hosts.

## Discussion

In this study, we present the first comprehensive depiction of bacteriophage integration in the human gut, as observed from the perspective of large-scale bacterial isolates. The highly uneven prophage distribution and the ultra-broad host range of integrated prophages in the human gut uncover potential diverse interaction patterns between bacteriophages and bacteria. The examination of antibiotic resistome and virulence factors has augmented our attention toward the future evaluation of the potential hazards associated with the transmission of antibiotic resistance and pathogenicity in the human gut microbiome. Furthermore, our identification of human gut-derived bacterial prophages also provided a substantial collection of superior reference sequences, which can be utilized for forthcoming investigations on the human gut phageomes.

Our findings suggest that a significant proportion, exceeding 90%, of the bacterial population in the human gut harbors prophage-like elements, but it is noteworthy that only a quarter of these bacteria possess complete prophages. The prevalence of incomplete prophage fragments could be attributed to adaptive evolution occurring within bacterial genomes. Host organisms employ various mechanisms, including genomic recombination, insertion, and deletion, to preserve beneficial functional genes and eliminate redundant genomes^24^ while also causing loss of the release function of prophages, thereby mitigating the risk of phages reentering the lytic cycle. The distribution of integrated prophages among different bacterial species exhibits notable diversity. The widespread presence of full-length prophages has been extensively reported in common pathogenic bacteria ,^6–11^ which typically inhabit various ecological niches; they may cause infections within the human body while also being capable of survival and proliferation in water, soil, or food. Bacteria occupying multiple ecological niches might face more selective pressures that exhibit a stronger tendency for prophage integration, thereby rapidly enhancing their adaptability in diverse environments.^25^ Similarly, the frequent prophage detection in lactobacilli is presumably due to their wide-ranging ecological distribution. Cornerstone species in the human gut, like *Bacteroides*, *Prevotella*, and *Bifidobacterium*, are largely constrained by ecological niches, ^26–28^ potentially indicating lower demands for prophages carrying functional genes. Additionally, we observed a lower likelihood of high-GC-content bacteria integrating prophages, likely due to the stronger hydrogen bonding within G-C base pairs compared to A-T base pairs, resulting in a more stable DNA chain structure and making it less susceptible to the integration of exogenous DNA fragments.

Previously, safety assessments of probiotic strains primarily involved antibiotic resistance, pathogenicity, metabolic characteristics, tolerance, and adverse reactions, with less focus on the associated risks of prophage integration within their genomes. Our research findings reveal that various lactobacilli strains commonly used as probiotic supplements not only frequently carry complete prophages but also exhibit instances of cross-genera integration. It has been demonstrated that *L. reuteri* in the human gut can release prophage particles at a high frequency under high fructose diet conditions.^29^ Consequently, when using lysogenic strains as probiotics, the potential induction and release of prophages they carry could pose risks of infection or gene transfer to other intestinal bacteria. We know that lactobacilli are generally one of the safest probiotics and rarely cause direct infections in the human body. However, the detected hemolysin genes within the prophages of multiple *L. paracasei* and *L. rhamnosus* strains in this study aroused our attention to their potential pathogenicity. We suggest continuous monitoring of the potential risks of lactic acid bacteria associated with prophage carriage and advocate for ongoing evaluation and regulation of their use in products to ensure their safety and efficacy.

Several human gut microbiota metagenomic analyses have indicated that the abundance of reads carrying mobile genetic elements harboring ARGs does not exceed 1% of the total viral reads.^30,31^ However, within our dataset, ~2.5% of the identified prophage genomes exhibited concealed potential ARGs, slightly surpassing estimations based on short-read metagenomic techniques. The possible reason for this is as follows: Currently, over half of the data stored in public repositories originates from common human bacterial pathogens. Although we have flattened the genomic data for each bacterial genus or species as much as possible, the extremely uneven prophage distribution, especially the detection of a large number of prophages in pathogens, has led to a biased higher detection rate of prophage-related ARGs in this study. Several cornerstone bacterial genera in the human gut, such as *Bacteroides* (0.3%), *Parabacteroides* (0.6%), *Prevotella* (0.8%), *Phocaeicola* (1.1%), and *Bifidobacterium* (1.3%), both presented relatively low detection rates of prophage-mediated ARGs in their genomes. Thus, our observations from the perspective of bacterial isolates reaffirm the traditional view that ARGs are rarely encoded in phages.^32^ Nevertheless, our extensive sampling data revealed that the probability of detecting prophage-associated ARGs exceeded 10% in multiple lactobacilli species, which is opposite to the conclusion (lactobacilli phages never carry ARGs) drawn by Tóth et al.^33^ using a limited dataset (579 isolates of 12 commonly employed probiotic bacterial species). Continual refinement of the transferable ARG dissemination network within the human gut microbiota is essential to guide research aimed at predicting and constraining the emergence of antibiotic resistance. Likewise, the detection rate (~5.8%) of toxin-related genes within prophages of human gut-origin bacteria might also be slightly overestimated due to the high prevalence of prophages among pathogenic bacteria. Besides directly eliciting inflammatory responses in human gut cells, certain toxins produced by gut bacteria target surrounding bacteria harboring temperate phages, arousing dormant prophages within these bacteria, consequently inducing bacterial lysis and leading to widespread alterations in the composition of the intestinal microbiota.^34^ Exploring whether gut bacteria gain a competitive advantage by acquiring prophages carrying toxin genes and how to prevent unintended harm to the human host due to such events warrants further in-depth investigation.

Through investigating the genomic homogeneity among prophage sequences, the linkages between prophages and human gut phageomes, and the infection records in bacterial CRISPR systems, we have determined the potential integration host range and infection host range for all prophages. This exploration concurrently provides insights into the role of phages in the human gut microbiome. As mentioned earlier, the observed incomplete prophage fragments are likely outcomes of bacterial domestication of phages, and these domesticated “Silencers” have become an integral part of bacteria. Those complete prophages that may have substantial autonomy in lysogenic integration and release display markedly distinct survival strategies in their protracted struggles with bacteria. Certain phages possess the ability to integrate into a wide range of host organisms but do not have any documented instances of infection within bacteria, which suggests that these “Hitchhikers” can establish a delicate coexistence with bacteria without causing lysis. This phenomenon lends support to the emergence of the “piggyback-the-winner” hypothesis^35^ in the human gut, where these phages adopt a temperate lifestyle in the intestinal microenvironment with high host density. In stark contrast, another subset of phages views bacteria as the prey to be slaughtered, leaving detectable infection records across an extensive range of bacterial hosts. Their lifestyle is more in line with the “Killer” role in the “Kill-the-winner” hypothesis.^36^ It is evident that more phages can only observe a limited range of host integration and infection, as well as leave multi-frequency infection records in the CRISPR system of a certain type of bacteria. In the protracted co-evolutionary process, bacteriophages and bacteria act as the “Red Queen”^37^ for each other. Bacterial hosts prevent phage invasion through diverse defense mechanisms, while phages can also evade bacterial defense systems by altering their DNA sequences. In summary, within the complex microbial ecosystem of the human gut, phages can probably choose the most suitable survival strategy based on their own and host characteristics.

At present, there is a divergence of opinions among researchers regarding the primary origins of bacteriophages in the human gut. Minot et al.^38^ reported that individuals who consume similar food items tend to possess more comparable gut phageomes, thereby implying that diet exerts the most immediate influence on phageome composition. Conversely, Breitbart et al.^39^ have discovered that the three most prevalent viruses found in the intestines of healthy infants are absent in both breast milk and formula, suggesting that diet may not serve as the primary source of intestinal virome. Another main viewpoint regarding the human gut phageomes indicates a predominance of temperate phages, particularly during early developmental stages.^40,41^ Nevertheless, our research findings do not support this hypothesis. We speculate that only a small fraction of prophages in the human gut are induced into independent extracellular phages, and they do not hold prominent positions within the human gut phageomes. Since Norman et al.^42^ reported that gut phageome changes are independent of bacteriome changes in patients with Crohn’s disease and ulcerative colitis, numerous investigations have commenced to investigate the interplay between phages and bacteria in the pathogenesis of specific ailments.^43–45^ Licznerska et al.^46^ proposed that the progression of the human body into a diseased state is accompanied by an augmented induction ratio of prophages, thereby influencing the balance between symbiotic and pathogenic bacteria and ultimately resulting in alterations in the composition of the gut microbiota. The extensive dataset of human gut-derived bacterial prophages obtained in this study holds significant potential as a valuable resource for forthcoming investigations. More in-depth knowledge about the complex link between gut phages and human diseases will hopefully come to light, along with new, effective ways to diagnose and treat these conditions.

Finally, it is imperative to acknowledge the limitations of this study. Although a substantial number of genomes were included, our genomic database only encompassed a limited fraction of the worldwide human gut bacterial diversity and focused solely on known prevalent gut symbiotic bacteria. Given the extensive diversity of unidentified bacterial genera within the human gut, our forthcoming research will endeavor to embrace a broader scope of bacterial genomes. This will allow us to construct a comprehensive landscape of the human gut-derived bacterial prophages, thereby facilitating a more intricate and comprehensive comprehension of the bacteria-phage cross-kingdom interactions. In addition, this study only indirectly evaluated the release and integration of prophages in the human gut through sequence alignments between the prophages and phageomes. In the future, we will prioritize investigating the inducibility and transformation potential of the predicted prophages, both on the bench and *in vivo*.

## Materials and methods

### Genome collection of human gut-derived bacteria

We first retrieved the GMrepo database^47^ (Data repository for human gut microbiota; accessed on July 15, 2022) for common bacterial species in the human gut (sample coverage ≥ 10%, average median abundance ≥ 0.01%) and also included temporarily unclassified bacterial species that frequently appeared (high-quality genome count ≥ 30) in the UHGG-MGYG database^48^ (a catalog of metagenomic assembled genes from the human gut microbiome; accessed on August 1, 2022). For these bacterial species, we collected available genomes (clearly identified as the source of human intestines/feces) from the NCBI GenBank database, the UHGG-MGYG database, and our laboratory. Our selection criteria for metagenomic-assembled genomes included a minimum genome completeness of 90% and a maximum contamination level of 5%. Furthermore, in cases where multiple genomes were accessible for a particular bacterial species, preference was given to genomes with higher Contig N50 values, greater genome completeness, and lower contamination levels.

Following data collection, we conducted a screening and taxonomy validation on the acquired bacterial genomes; specifically, we eliminated individuals within the same species that exhibited substantial deviations in genome lengths. The pairwise average nucleotide identity (ANI) values were determined using the PYANI v. 0.2.9 package,^49^ and any genomes significantly divergent from the established standard (with a threshold of 94%) were excluded. It is important to highlight that, although taxonomic uncertainties and considerable nucleotide variations were observed among *Faecalibacterium prausnitzii* genomes, we still treated it as a bacterial species before a clear taxonomic definition. All bacterial taxonomic names utilized in this investigation adhered to the updated nomenclature requirements updated by NCBI. Ultimately, our study compiled a comprehensive collection of 43,942 bacterial genomes derived from the human gut (37,629 from the UHGG-MGYG, 5,523 from the NCBI, and 790 from our laboratory). These bacteria are classified into 439 distinct species, 188 genera, 65 families, 34 orders, 20 classes, and 12 phyla (Figure 1a). Among these, there were 194 species with an abundance of over 100 available genomes, 161 species with 50 to 100 genomes, 31 species with 30 to 50 genomes, and 53 species with fewer than 30 genomes. Detailed information regarding the bacterial species involved in this study can be found in Supplementary Table S1.

### Prophage prediction and integrity assessment

The PHAge Search Tool enhanced release (PHASTER; http://phaster.ca/)^50^ was employed as a predictive tool for the identification of prophages in bacterial genomes derived from the human gut. According to the software’s specifications, a scoring criterion was applied to each predicted prophage region.^51^ If a predicted prophage region fully encompassed a specific phage organism in the database, it received a total score of 150. Alternatively, if this condition was not met, two additional methods were utilized: (1) The total score of the predicted prophage region is determined by calculating the ratio of coding sequences (CDS) in the region that match those of a specific phage, multiplying this ratio by 100, and calculating the query coverage of the region with the target phage, which is then multiplied by 50. The sum of these two items constitutes the total score. (2) In the event that any of the designated phage-related genes, such as integrase, transposase, protease, terminase, portal, capsid, head, tail, fiber, coat, plate, or lysin, were identified, the score was augmented by 10 for each gene detected. If the predicted prophage region met the following criteria: a genome size of 30 Kb, a CDS number of 40, and a proportion of phage-related proteins and hypothetical proteins of 70%, the score was incremented by 10 for each fulfilled criterion. The final score of the region was determined by comparing the total scores of the two methods, with the higher score being selected. A region with a score below 70 was designated as an incomplete prophage; a region with a score ranging from 70 to 90 was classified as a questionable prophage; and a region with a score of 90 or higher was identified as an intact prophage. Among all 105,613 prophage regions predicted by PHASTER, 12.5% (*n* = 13,161) of them are intact prophages, 10.6% (*n* = 11,204) are questionable, and most of them (72.9%, *n* = 81,248) were marked as incomplete prophage fragments (Figure 1a).

The completeness level of all predicted prophage regions was evaluated using the software CheckV v. 1.0.1^52^ with default parameters and databases. As shown in Figure 1a, 1.0%, 7.1%, and 15.8% of all 105,613 prophages were identified as complete, high-quality, and medium-quality, respectively; another 55.6% and 20.6% were labeled as low-quality and not-determined. In this study, prophages identified as intact by PHASTER or as complete/high-quality by CheckV were considered complete prophages (Supplementary Table S3).

### Clustering of prophages, construction of phylogenetic trees, and functional gene annotation

The clustering of all 105,613 prophages was carried out utilizing the built-in algorithm Linclust^53^ in MMseqs2^54^ (–min-seq-id 0.9 –cov-mode 1 -c 0.9 –cluster-mode 2 –max-seq-len 1,000,000). In cases where prophages produced a sufficient alignment (>90% identity and > 90% query coverage) with one another, only the longer sequence was retained in the dataset. The resulting dataset comprised 47,965 representative prophages. The construction of the phylogenetic tree was executed using phyML v. 3.1,^55^ employing the neighbor-joining method and based on the entire prophage genomes. The prediction of open reading frames (ORFs) within the phage genome was accomplished using Prodigal v. 2.6.3,^56^ followed by annotation using BLASTp v. 2.14.0^57^ against the Non-Redundant Protein Sequence Database of NCBI.

### Acquisition of the CRISPR-spacers and alignment with prophages

The web server CRISPRCasFinder^58^ (https://crisprcas.i2bc.paris-saclay.fr/) was utilized to predict CRISPR systems among all 43,942 bacterial genomes in this study and obtain a total of 933,632 spacer sequences. In addition, we also collected 1,846,441 spacers from the MGV, together forming the CRISPR-spacer dataset used in this study. Then, we performed BLASTn alignments between all prophages and the CRISPR-spacer dataset. If a spacer generated an exact match (identity = 100%, coverage = 100%) with a prophage, we assigned the bacterial host associated with the spacer to the phage.

### Prediction of ARGs and virulence factors among prophage genomes

The ORFs of all prophage genomes were compared to the latest version of the Comprehensive Antibiotic Resistance Database^59^ using the Resistance Gene Identifier (RGI) v. 5.1.0 to identify potential ARGs (the strict model with default parameters) and were aligned with the Virulence Factors Database^60^ (http://www.mgc.ac.cn/VFs/) using BLASTp to identify putative virulence factors with a threshold of identity ≥ 30% and coverage ≥ 70%.

### The linkages between human gut-derived bacterial prophages and human gut phageome contigs

The publicly available reference phage sequence database used in this study was gathered from the GPD^17^ (https://ftp.ebi.ac.uk/pub/databases/metagenomics/genome_sets/gut_phage_database/; 142,809 sequences; accessed on May 29, 2021), the MGV v. 1.0^18^ (https://portal.nersc.gov/MGV/; 189,680 sequences; accessed on July 8, 2021), and virus genomes in the NCBI Nucleotide Sequence Database (nt_viruses; https://www.ncbi.nlm.nih.gov/; 2,266,959 sequences; accessed on July 15, 2021). The pairwise alignments of human gut-derived bacterial prophages with the reference phages were conducted using BLASTn v. 2.13.0. If a prophage generated sufficient similarity (identity ≥50%; query coverage ≥ 90%) with any sequence in the reference phage database, it was classified as a known phage; otherwise, it was categorized as a newly discovered phage.

The human gut phageome data from Irish, Dutch, and Chinese studies used in this study were obtained from Shkoporov et al.^61^ (https://doi.org/10.6084/m9.figshare.9248864.v1), Garmaeva et al.^62^ (https://doi.org/10.6084/m9.figshare.12666830), and our previous research (https://doi.org/10.5281/zenodo.10125150). These three studies employed a standardized approach to acquire extracellular free phage sequences within the human intestinal tract. The brief steps are as follows: initial enrichment and purification of free viral particles from fecal samples, followed by DNA sequencing via high-throughput techniques, and ultimately culminating in the acquisition of phageome data through a combination method of *de novo* assembly and virome identification bioinformatics. The host assignments for human gut phageome contigs at the bacterial genus level were carried out in the following steps: Initially, if either of the phageome contigs exhibited a substantial alignment (identity ≥50%; query coverage ≥ 90%) with an annotated phage present in the reference database, the bacterial host associated with the known phage was assigned to the respective phageome contig. Subsequently, BLASTn alignments were performed between all human gut phageome contigs and the CRISPR-spacer database (1,846,441 spacers, https://portal.nersc.gov/MGV). If a phageome contig displayed an exact match (identity = 100%, coverage = 100%) with a spacer, the corresponding bacterial host was assigned to it. Finally, all phageome contigs were aligned with the non-redundant prophages identified in this study, and contigs that yielded sufficient comparisons (identity ≥50%; query coverage ≥ 90%) with prophages were assigned corresponding hosts.

### Data visualization and statistical analysis

The Nightingale rose diagrams, combining pie charts, Sankey diagrams, circle diagrams, advanced heatmap plots, doughnut charts, violin plots, and bean plots were visualized using the OmicStudio tools at https://www.omicstudio.cn. The bubble charts were visualized using the ImageGP at https://www.bic.ac.cn/BIC/#/. The phylogenetic trees were visualized using the webserver iTOL v.6.8.1 at https://itol.embl.de/. The bar graphs, stacked histograms, and boxplots were generated using GraphPad Prism v8.0. The network graphs were displayed using the software Gephi v0.10.1. The genome organization of the representative phage was visualized using the R package gggenes. Some figure elements used in this study were obtained from the Figdraw in Home for Researchers (www.home-for-researchers.com/). The non-parametric tests (the Mann-Whitney U test and the Kruskal-Wallis test) were conducted using SPSS PASW Statistics v18.0.

## Supplementary Material

Supplementary_Table_S1_S9.xlsx

Supplementary_Figure_S1_S4.docx

## Data Availability

The specific information and accession numbers of the bacterial genomes used in this study (Data S1), the sequences of all human gut-derived bacterial prophage sequences (Data S2), non-redundant representative prophages (Data S3), and CRISPR-spacers (Data S4) obtained in this study, as well as the analysis process file of human gut phageomes (Data S5) are available at the Zenodo repository under https://doi.org/10.5281/zenodo.10258953. Any additional information required to reanalyze will be made available upon request.
