# Supplementary material for: A universe of human gut-derived bacterial prophages: unveiling the hidden viral players in intestinal microecology
Source: Gut Microbes. 2024 Feb 1;16(1):2309684. doi: 10.1080/19490976.2024.2309684 (PMC10841027; doi:10.1080/19490976.2024.2309684)
Supplement: Supplementary_Figure_S1_S4.docx [file KGMI_A_2309684_SM0045.docx]

**Supplemental Figure S1-S4**

**A universe of human gut-derived bacterial prophages: Unveiling the hidden viral players in intestinal microecology**

Zhangming Pei, Yufei Liu, Yutao Chen, Tong Pan, Xihao Sun, Hongchao Wang, R. Paul Ross, Wenwei Lu, Wei Chen

**
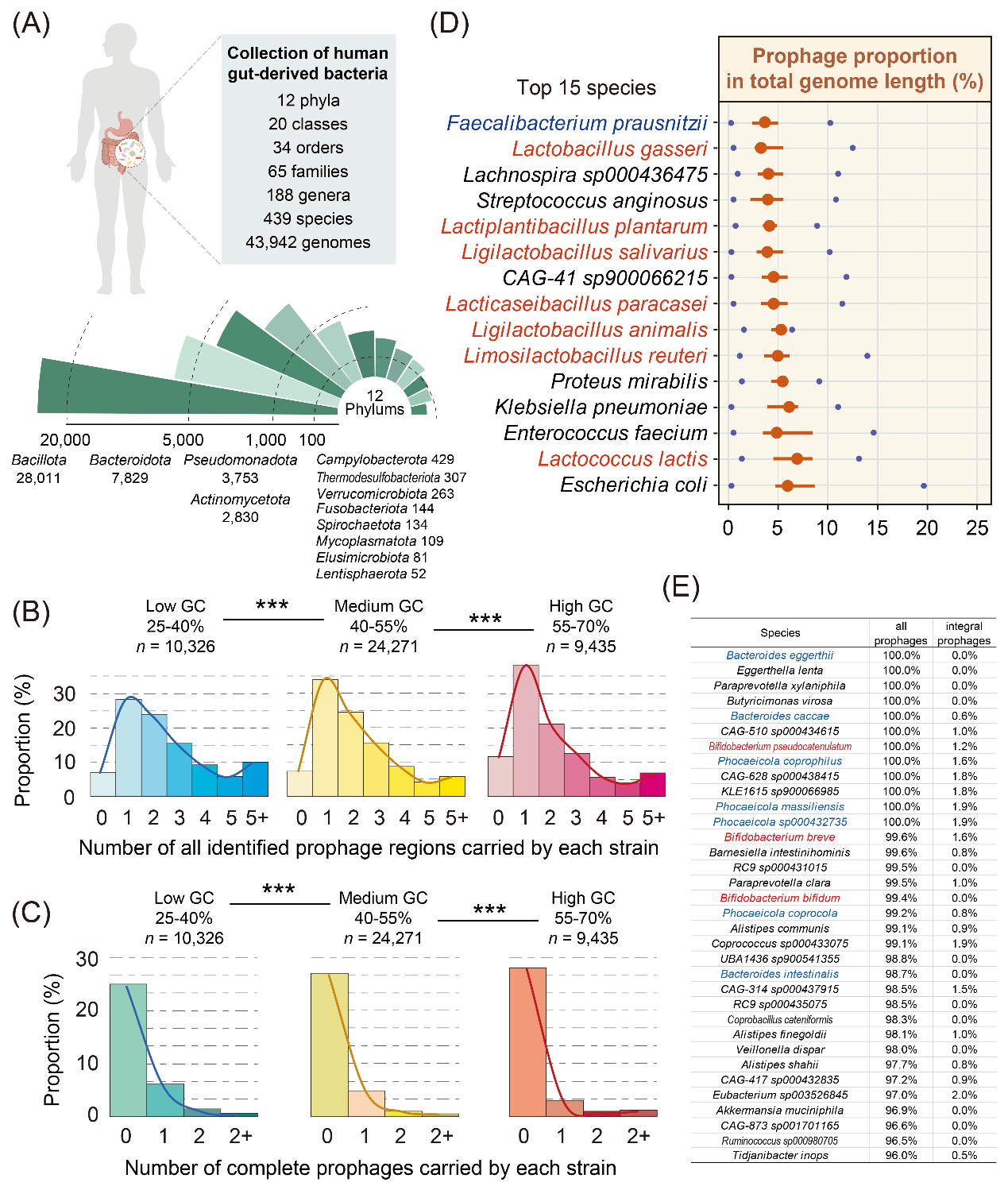
Fig. S1. The overall landscape of human gut-derived bacterial prophages (related to Fig. 1). (A)** The collection of human gut-derived bacteria comprises 43,942 genomes, representing 12 phyla and 439 distinct species. **(B)** Comparison of the number of all prophage regions carried by the “Low GC”, “Medium GC”, and “High GC” bacteria. **(C)** Comparison of the number of complete prophages carried by the “Low GC”, “Medium GC”, and “High GC” bacteria. Statistical significance tests were performed using the nonparametric Mann-Whitney U test, and the two-tailed *p* values were calculated. ***: *p* < 0.001. **(D)** Top 15 bacterial species with the highest content of prophages. The leftmost and rightmost blue dots represent the minimum and maximum values, respectively. The orange dot and line segment represent the median and the interquartile distance, respectively. The specific data for each bacterial species is listed in Supplementary **Table S4**. **(E)** Bacterial species with high-frequency detection of prophage fragments but rare integral prophages.


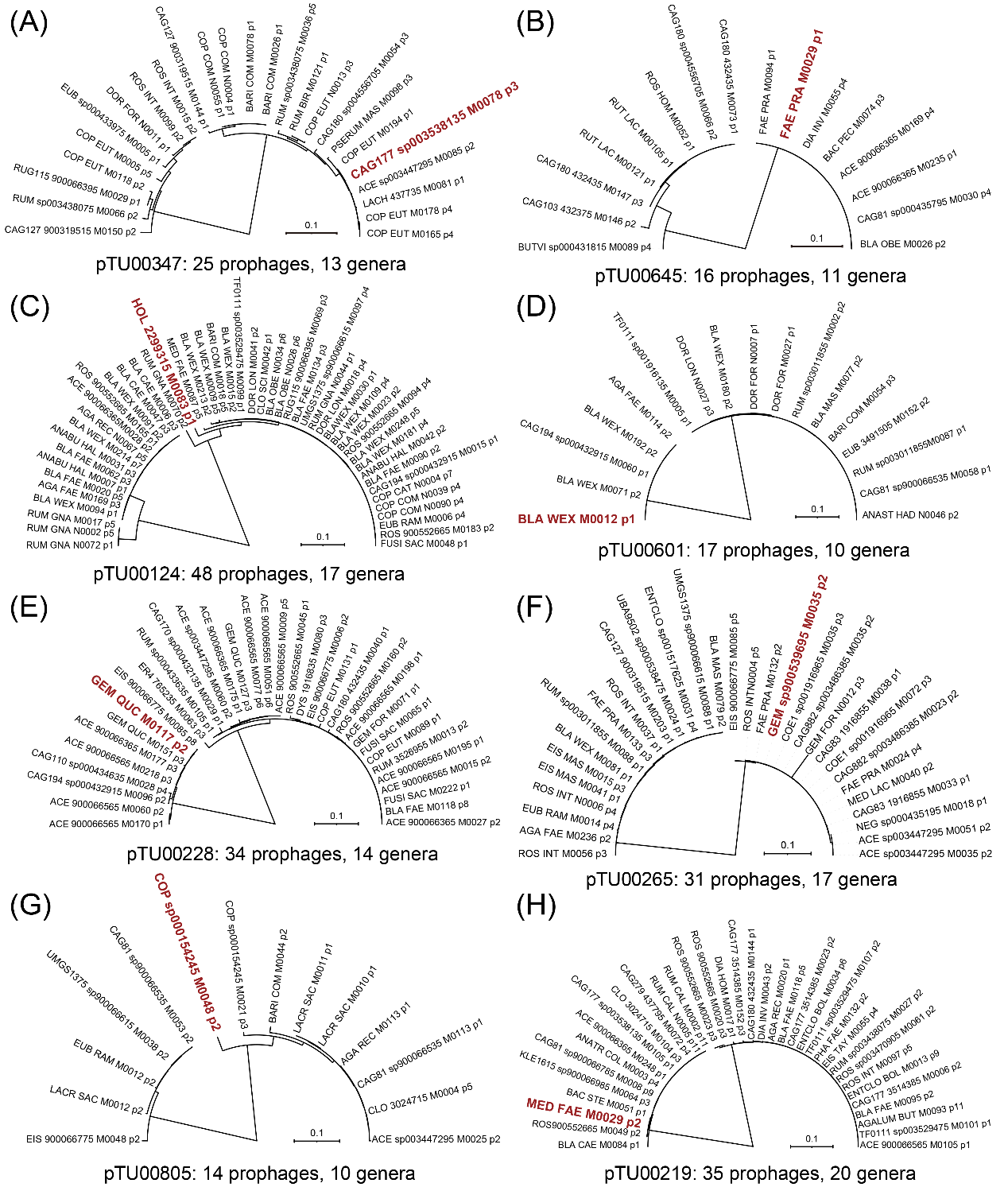


**Fig. S2.** Clustering and phylogenetic trees of eight ultra-broad-spectrum integrated prophages. **(A)** pTU00347, CAG177_sp003538135_M0078_p3. **(B)** pTU00601, FAE_PRA_M0029_p1. **(C)** pTU00228, HOL_2299315_M0083_p1. **(D)** pTU00265, BLA_WEX_M0012_p1. **(E)** pTU00228, GEM_QUC_M0117_p2. **(F)** pTU00265, GEM_sp900539695_M0035_p2. **(G)** pTU00805, MED_FAE_M0029_p2. **(H)** pTU00219, COP_sp000154245_M0048_p2.


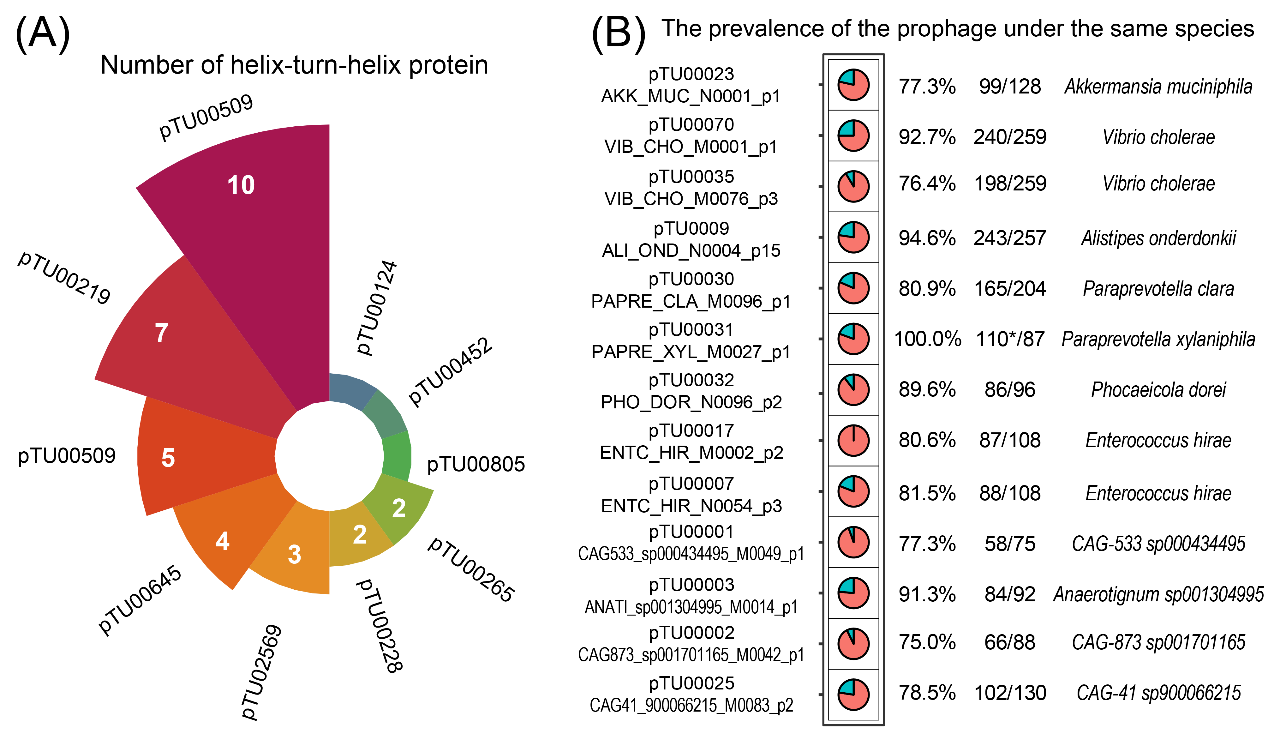


**Fig. S3. (A)** The number of helix-turn-helix proteins detected in 10 ultra-broad-spectrum integrated prophages. **(B)** The 13 prophages are highly prevalent (> 75% of strains) within the same bacterial species.


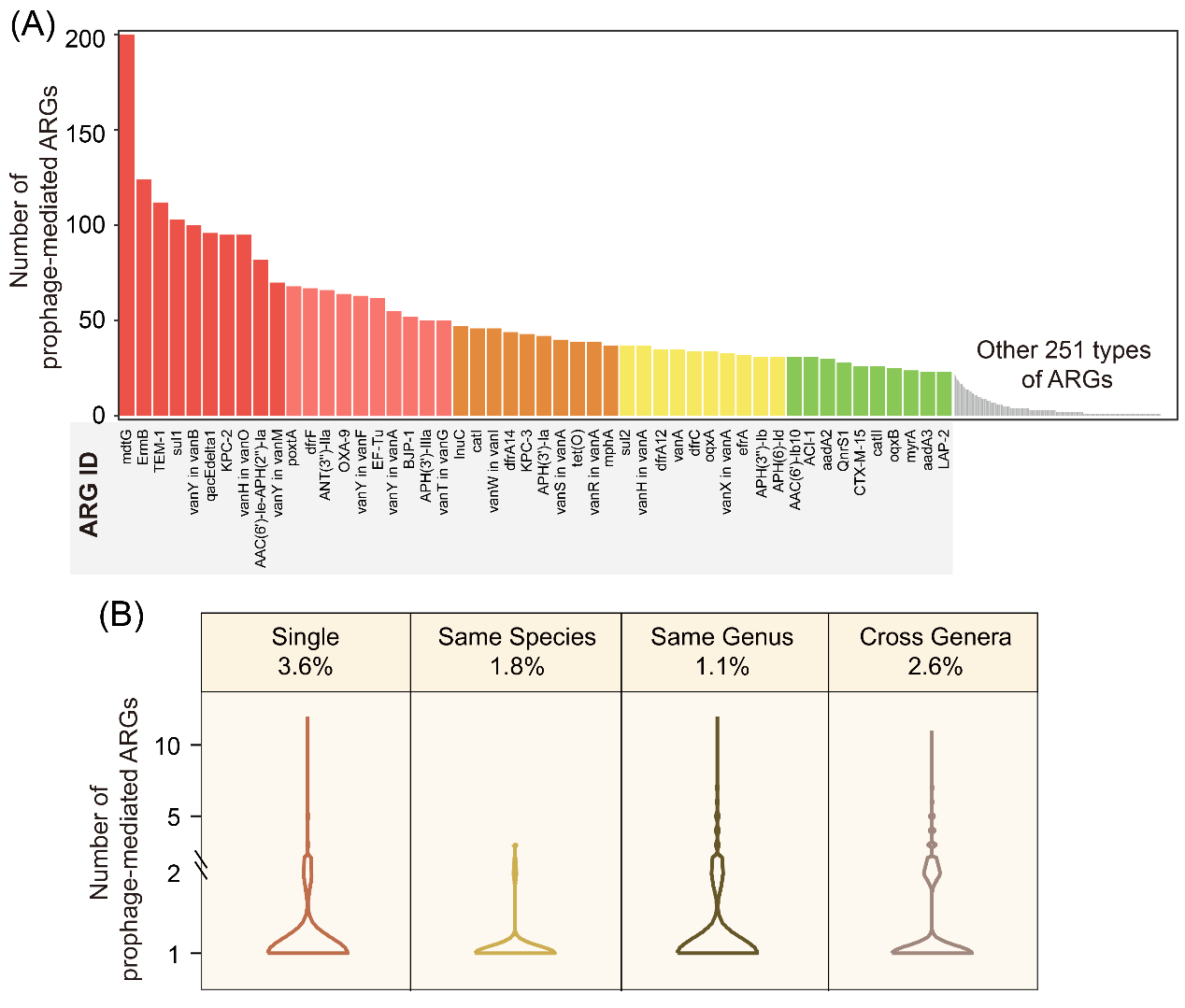


**Fig. S4. (A)** The detected quantity of 301 ARGs in all prophages. **(B)** The detection frequency of ARGs in prophages with different integration host ranges and the distribution of the number of ARGs carried by each prophage.
